# On the Treatment of Asthma

**Published:** 1867-08

**Authors:** W. Anderson

**Affiliations:** Pontiac, Ill.


### ON THE TREATMENT OF ASTHMA.

By W. Anderson, M.D., Pontiac, Ill.

Editor Medical Journal:

*Dear Sir:*—Please permit me to offer to your readers a few practical remarks on the subject of Asthma, the disease universally and improperly, by the unprofessional, called phthisic, a fact at which we should not demur while the profession adheres to so many barbarous and uncouth, not to say unscientific, names.

This, of all diseases, requires the most strictly tentative treatment, and by adhering too closely to certain remedies in all cases, strikes me as a reason of so little success amongst physicians in its treatment; though I admit it is not curable, and all treatment for this disease is strictly palliative. Or, rather, I would say, while the disease can generally be mitigated and the paroxysms often cut short, I would not pretend to claim that the affection can be eradicated from the system. What may greatly benefit one sufferer may only oppress the next, and not avail in one case out of the next five. The physician should try, and keep on trying, the various remedies, in quick succession, till the right one is found. Whoever prescribes according to authorities, without trying a diversity of remedies, will generally furnish his patient no relief. The patient will generally know what is best adapted to his particular case, and should be thoroughly consulted.

This disease is sometimes brought on by menstrual obstruction. This must be rare, as I am not aware that it has ever been noticed by medical authors. I was recently called to see a case of this kind. She had been troubled at times since the age of puberty; nothing would give relief but bleeding or dry-cupping over the lungs, either on the breast, side, or back. That the paroxysms were caused by a reflux of blood to the lungs was evident. It could not have been merely from the retention in the system of an exciting material, for then bleeding or cupping would not avail. She could get relief in no other way, and these never failed. She always had a paroxysm following *obstruction from whatever cause.* I never knew blood-letting to avail in any cases but the above.

It is well known that heart-diseases sometimes result from asthma. In such cases, the ordinary remedies for heart-disease, veratrum, aconite, digitalis, etc., cannot be taken, and should not be given, as they cause smothering and are dangerous and useless.

